# Anlotinib Combined With Chemotherapy for Recurrence of Pulmonary Sarcomatoid Cancer Previously Surgically Treated: A Case Report and Literature Review

**DOI:** 10.3389/fonc.2021.639168

**Published:** 2021-05-11

**Authors:** Jing Li, Hejun Liang, Jian He, Xin Sui, Yanru Qin

**Affiliations:** Department of Oncology, The First Affiliated Hospital of Zhengzhou University, Zhengzhou, China

**Keywords:** pulmonary sarcomatoid cancer (PSC), anlotinib, chemotherapy, targeted therapy, case report

## Abstract

**Background:**

Pulmonary sarcomatoid cancer (PSC) is a very rare subtype of poorly differentiated non-small-lung-cancer (NSCLC) with very poor prognosis. To date, the optimal treatment for PSC has not been elucidated, and the efficacy of anlotinib in PSC has not been previously reported.

**Case Presentation:**

A 77-year-old male patient was admitted with cough, expectoration, and blood-stained sputum for one month. CT showed a soft mass in the inferior lobe of the right lung, which was diagnosed as spindle cell carcinoma (PSC) by histopathology. A videothoracoscopic right lower lobectomy and mediastinal lymph node dissection procedure was performed on the patient, but the disease recurred one month after surgery. The patient was then given first-line chemotherapy with gemcitabine and albumin paclitaxel for one cycle, but the disease continued to progress. The patient then received anlotinib combined with second-line chemotherapy (dacarbazine and cis-platinum) for six cycles, and the response reached complete remission (CR). Then the patient was given maintenance therapy with anlotinib alone, and the disease was still stable at the most recent reexamination. Progression-free survival (PFS) has lasted for more than two years, without any intolerable toxicity.

**Conclusion:**

This postoperative recurrent PSC patient achieved significant clinical benefits with anlotinib treatment. Our findings provide direct evidence of the efficacy of anlotinib in PSC. More studies are needed to confirm our observation.

## Introduction

Sarcomatoid carcinoma (SC) is a rare malignancy with a combination of epithelial and sarcoma or sarcoma-like components (1, 2). Pulmonary sarcomatoid carcinomas (PSCs) are a heterogeneous group of non-small-cell lung carcinomas containing five subgroups, namely pleomorphic carcinoma, spindle cell carcinoma, giant cell carcinoma, carcinosarcoma, and pulmonary blastoma, which comprise 0.1% to 0.4% of all pulmonary malignancies (3). This malignancy has been reported to have very poor prognosis and shorter overall survival than other NSCLCs (4). Epidemiologically, the average age of diagnosis is 60 years. Because SC is most commonly observed in heavy smokers, there is also a male predominance (5). PSCs have been shown to respond poorly to platinum-based chemotherapy, and thus there is an urgent need for new and effective therapeutic options (6).

Anlotinib hydrochloride is a novel multitarget tyrosine kinase inhibitor (TKI) that has a significant inhibitory effect on tumor angiogenesis and proliferative signaling by selectively targeting VEGFR-2,-3 and FGFR-1,-2,-3,-4 and suppressing the activity of PDGFRα/β, C-has Kit, Ret, Aurora-B, C-FMS, and discoidin domain receptor (DDR1) (7). In preclinical studies, anlotinib has shown significant antitumor activity against a variety of xenograft models (7). In a phase III study called ALTER 0303, anlotinib showed a significant increase in OS and PFS in patients with advanced NSCLC as a third or later line of treatment (8). In a phase II study for patients with refractory soft-tissue sarcoma (STS), anlotinib also showed remarkable antitumor activity; overall, the 12-week progression-free rate (PFR_12 weeks_) and the overall response rate (ORR) were 68% and 13%, respectively, and the PFS and overall survival (OS) were 5.6 months and 12 months, respectively (9).

Here, we present a case report that showed an excellent therapeutic effect of anlotinib-combined chemotherapy against this malignancy.

## Case Presentation

**Figure 1:**
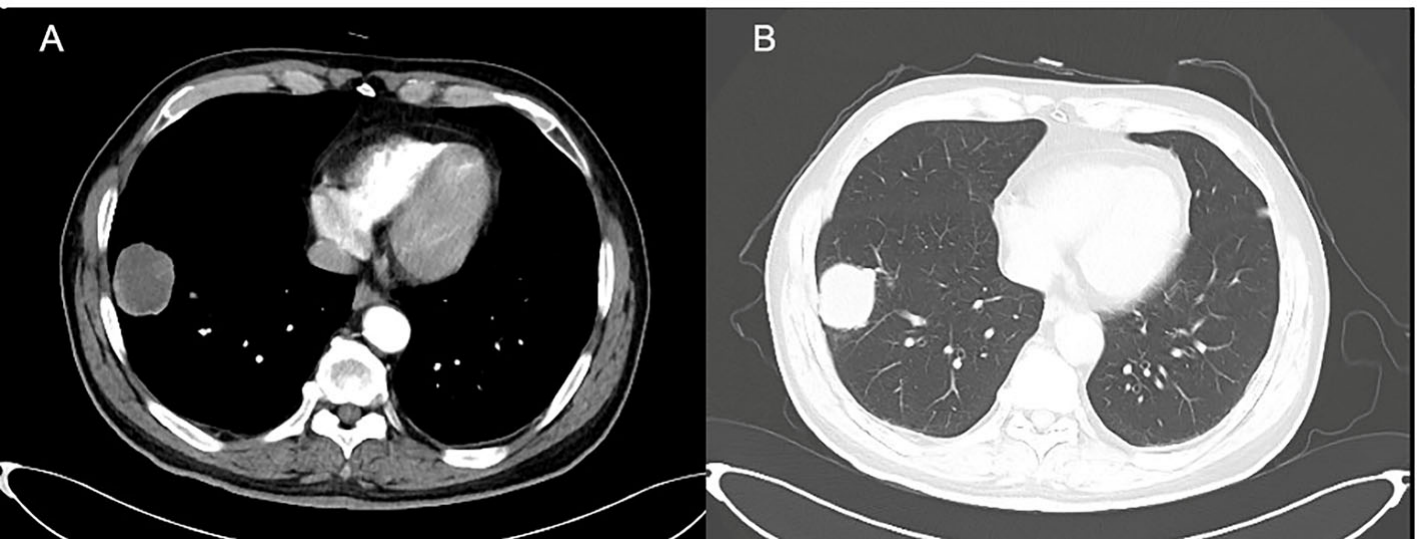
Occupation in the right inferior lobe of lung. **(A)** lung window, **(B)** mediastinal window.

**Figure 2:**
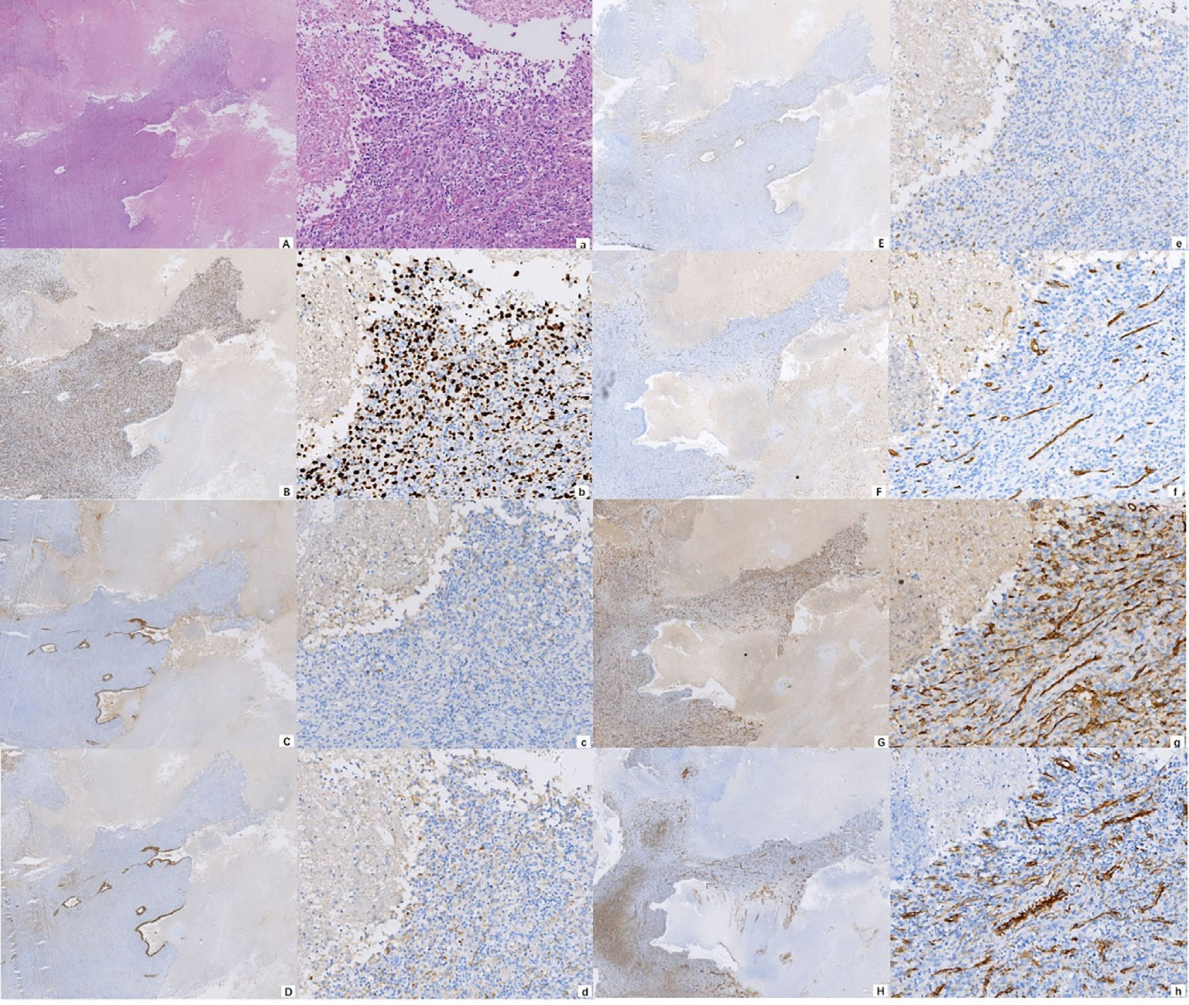
Photomicrograph showing the cytomorphological finds **(A)** H&E ×10; a H&E ×100) and immunohistochemical staining of the malignancy, positive for Ki-67 **(B)** IHC ×10; b IHC ×100), EMA **(C)** IHC ×10; c IHC ×100), CK8/18 **(D)** IHC ×10; d IHC ×100), TTF-1 **(E)** IHC ×10; e IHC ×100), CD34 **(F)** IHC ×10; f IHC ×100), CD31 **(G)** IHC ×10; g IHC ×100), and SMA **(H)** IHC ×10; h IHC ×100).

**Figure 3:**
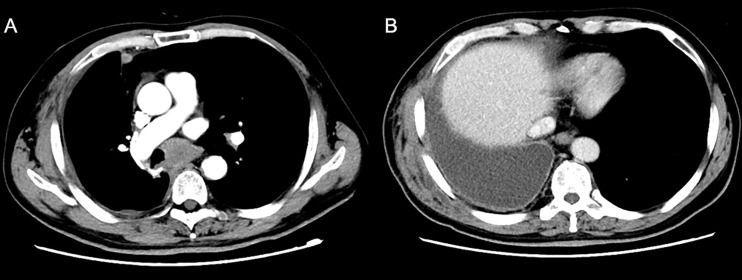
A reexamination CT scan one month after surgery showed new soft tissue nodules in the mediastinum.

**Figure 4:**
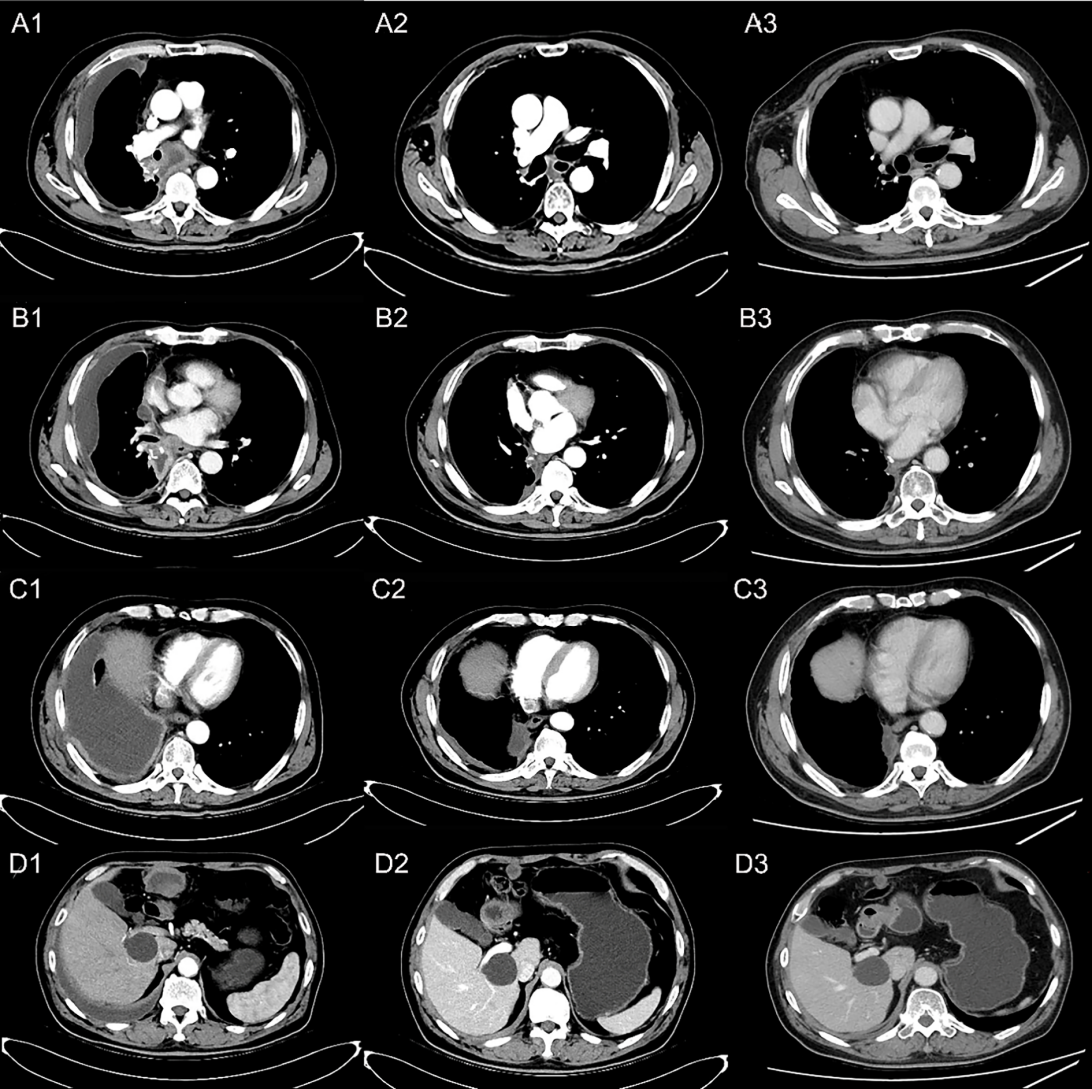
Reexamination results showing tumor regression by contrast-enhanced CT scan. **(A)** Occupation in mediastinum; **(B)** thickening of the right pleura; **(C)** soft tissue by the side of the right bronchial stump; **(D)** metastatic lesion on the right upper abdomen. (A1, B1, C1, D1) PD after one cycle of first-line chemotherapy; (A2, B2, C2, D2) disease PR after two cycles of the second-line treatment; (A3, B3, C3, D3) disease remaining as PR after four cycles of the second-line treatment.

**Figure 5:**
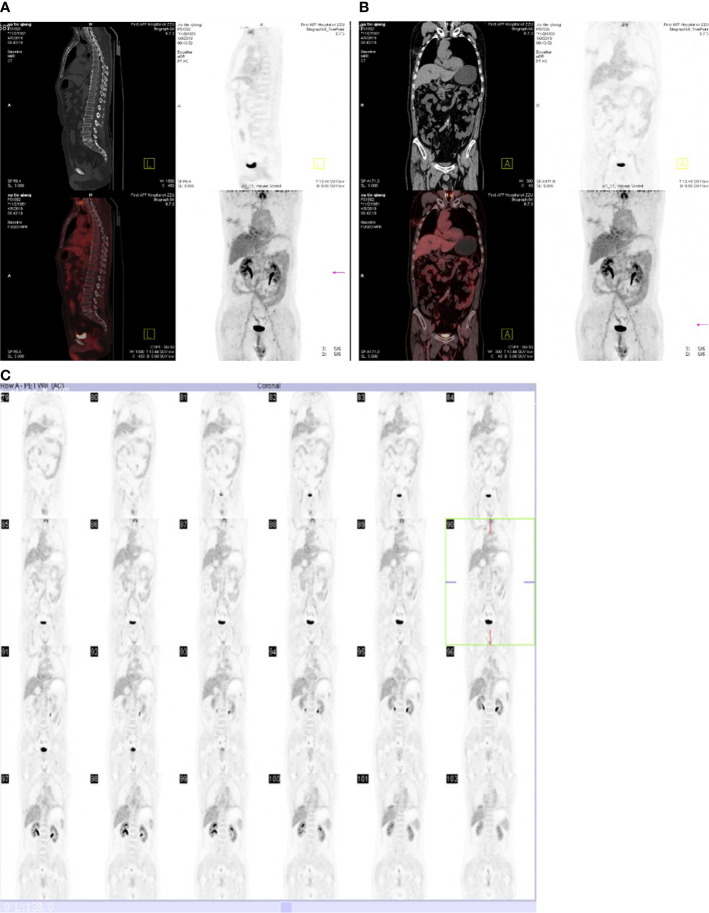
Reexamination PET-CT scan showing CR after six cycles of second-line treatment.

A 77-year-old male was admitted to our hospital complaining of coughing, expectoration, and blood-stained sputum for one month on September 11, 2018. There were no symptoms of fever, shortness of breath, chest stuffiness, or chest pain. He had a history of smoking for 40 years and smoked up to 20–25 cigarettes each day. In addition, he also had a history of stage 3 hypertension for 20 years and nifedipine delayed-release tablets were used to control blood pressure. Blood chemistry and tumor markers were within normal limits. A contrast-enhanced CT scan of the chest and abdomen showed a soft mass (42 × 38 mm) in the inferior lobe of the right lung without any lymph nodes or distant metastasis (**Figure 1**). A videothoracoscopic right lower lobectomy and mediastinal lymph node dissection procedure were performed on the patient on August 3, 2018. The postoperative pathological examination revealed a pulmonary sarcomatoid carcinoma with a size of 3.2×2.5×1.5 cm and with negative surgical margins of the bronchus and vessels. The subtype of PSC was confirmed as spindle cell carcinoma by the pathologist. A total of 37 lymph nodes were retrieved, including the second, fourth, seventh, tenth, and eleventh groups of lymph nodes, and all of them were negative. The TNM staging was T2aN0M0 IB according to the 8^th^ edition of the AJCC/UICC TNM staging system for lung cancer. Immunohistochemistry showed that the cells were stained positive for EMA(focal+), CK8/18(focal+), TTF-1(focal+), Ki-67(70%), CD34(focal+), CD31(+), and SMA(+), and negative for AE1/AE3(−), NapsinA(−), CK5/6(−), P40(−), P63(−), S-100(−), ERG(−), and Desmin(−) (**Figure 2**). Next-generation sequencing (NGS) showed 10 somatic mutations in BRAD1 [p.E355K(c.G1083A)], EPCAM [p.F20S(c.T59C)], EPHA3 [p.R274Q(c.G821A)], FGFR2 [p.A389V(c.C1166T)], KRAS [p.G12C(c.G34T)], RAC1 [p.A59G(c.C176G)], RAD54L [p.R688C(c.C2062T)], SMRCA4 [p.A733V(c.C2198T)], TERT [c.C-124T], and TP53 genes [p.R80K(c.G839A)]. Nevertheless, common mutations such as in EGFR, ALK, and ROS-1 genes were not found by NGS. The tumor mutational burden (TMB) was 9.7 mutations/Mb and the microsatellite status was microsatellite stability (MSS). Unfortunately, follow-up CT reexamination one month after surgery demonstrated disease recurrence in the mediastinum and right pleural effusion (**Figure 3**). Then the patient underwent one course of first-line chemotherapy consisting of gemcitabine and albumin paclitaxel. However, the coughing and expectoration persisted, and there was no significant relief after discharge. Because new symptoms of chest stuffiness and fatigue increased gradually, the patient was readmitted for a CT scan, which showed that the tumor had progressed in the mediastinum, the right pleura, and bronchial stump, and there was a new distant metastasis in the upper abdomen (**Figures 4**–A1, B1, C1, D1). The response evaluation was progressive disease (PD). Therefore, we changed the regimen to anlotinib combined with second-line chemotherapy consisting of dacarbazine and cis-platinum. After one cycle of treatment, symptoms of coughing, expectoration, chest stuffiness, and fatigue were significantly relieved. After two cycles of treatment, the symptoms disappeared substantially. Follow-up CT reexamination after two cycles of treatment showed that the tumor regressed significantly and the response evaluation was partial remission (PR) (**Figures 4**–A2, B2, C2, D2). After four cycles of treatment, a CT scan demonstrated that the tumor remained in remission, and the response evaluation remained PR (**Figure 4**–A3, B3, C3, D3). PET-CT was performed for therapeutic response assessment, and the results showed CR after the completion of six cycles of treatment (**Figure 5**). Subsequently, the patient continued taking anlotinib as a maintenance treatment. In the most recent reexamination (more than two years after therapy), the disease was still stable, and there was no intolerable toxicity.

## Discussion

PSC is a rare malignancy of NSCLC and has a poorly differentiated and aggressive signature (10). At present, there are no standard treatments for the malignancy. Platinum-based chemotherapy for NSCLC has long been used for PSC patients, but prognosis has remained poor and the median OS is no more than nine months for metastatic disease according to previous studies (4). In previous clinical practice, we treated some PSC patients using platinum-based chemotherapies such as pemetrexed combined with cisplatin or gemcitabine combined with cisplatin, but the responses were disappointing. We also used gemcitabine and taxanes, which are therapeutic regimens for STS. Unfortunately, the response has still been unsatisfactory. Therefore, it is necessary to identify new active agents to improve the therapeutic effect for PSC patients. Sarcomatoid carcinoma is a malignancy with a combination of epithelial and sarcoma or sarcoma-like components. We therefore used a new chemotherapy agent comprising dacarbazine and cis-platinum. Dacarbazine is a standard anti-sarcoma agent with classically known activity, generally reserved as a later line of therapy, and cis-platinum is a major component of NSCLC treatment (11, 12). Eventually, the disease achieved CR after six cycles of the second-line treatment.

The programmed death-1 (PD-1) and programmed death ligand-1 (PD-L1) axis is a classical immune checkpoint pathway. Tumor cells utilize this immunosuppressive co-signal to evade immune defenses in the tumor microenvironment. A blockade of the PD-1/PD-L1 axis has restored effector T-cell function and enhanced antitumor immune activity in preclinical studies (13). Consistently, clinical trials of PD-1/PD-L1 signal-blockade agents have shown prominent antitumor efficacy in several malignancies including NSCLC, and the outcome is positively related to the expression level of PD-1 (14–16). With regard to PSC, previous studies have shown that the expression level of PD-L1 is high (17). Therefore, we tested PD-L1 expression in this patient. However, as the test results were negative, a PD-1 inhibitor was not used.

Recently, several studies have focused on the genetic features of this disease and revealed a wide range of alterations, including TP53, EGFR, KRAS, PIK3CA, STK11, BRCA1/BRCA2, and IDH1 mutations; MET exon 14 mutations; and MET, ALK, EGFR, CDK4, and MDM2 amplifications, some of which are associated with targeted therapy through the MET receptor pathway or MAP-kinase pathway (3, 6). In this case, ten somatic mutations including two common mutations, specifically TP53 and KRAS, along with other occasional mutations, specifically BRAD1, EPCAM, EPHA3, FGFR2, RAC1, RAD54L, SMARCA4, and TERT, were found by NGS. Unfortunately, common mutations of EGFR, ALK, and ROS-1 genes were not found.

Anlotinib is a multitargeting TKI that can significantly inhibit tumor proliferation, vasculature, and the tumor microenvironment by selectively targeting VEGFR-2,-3, FGFR-1,-2,-3,-4, PDGFRα/β, C-Kit, Ret, Aurora-B, c-FMS, and DDR1 (7). A phase I study showed that anlotinib has promising anti-tumor potential against a variety of tumors, including STS and NSCLC (18). At present, anlotinib has been approved as a second- or later-line treatment for STS and third- or later-line treatment for NSCLC based on the ALTER 0203 and ALTER 0303 clinical trials in China (8, 9). Considering that NGS for this patient revealed a few gene mutations, including of the FGFR gene, we combined chemotherapy with anlotinib, which selectively targets FGFR, and achieved prominent tumor regression and a long PFS without evident toxicity.

## Conclusion

In summary, we combined chemotherapy with a small molecular TKI known as anlotinib for a PSC patient and achieved a long PFS and remarkable tumor regression. To our knowledge, this is the first case report of the successful treatment of this disease by using this combined treatment. Future clinical trials are expected to validate these new treatments for PSC patients.

## Data Availability Statement

All datasets generated for this study are included in the article.

## Ethics Statement

Studies involving human participants were reviewed and approved by the Ethics Committee of the First Affiliated Hospital of Zhengzhou University of Henan Province. The patients/participants provided their written informed consent to participate in this study. Written informed consent was obtained from the individual(s) for the publication of any potentially identifiable images or data included in this article.

## Author Contributions

JL: treatment of the patient, clinical data collection, and manuscript preparation. HL: literature research and manuscript preparation. JH: treatment of the patient and clinical data collection. XS: treatment of the patient and clinical data collection. YQ: treatment of the patient and guarantee of the integrity of the whole research process. All authors contributed to the article and approved the submitted version.

## Conflict of Interest

The authors declare that the research was conducted in the absence of any commercial or financial relationships that could be construed as a potential conflict of interest.
